# Automatic Setting Procedure for Exoskeleton-Assisted Overground Gait: Proof of Concept on Stroke Population

**DOI:** 10.3389/fnbot.2018.00010

**Published:** 2018-03-19

**Authors:** Marta Gandolla, Eleonora Guanziroli, Andrea D'Angelo, Giovanni Cannaviello, Franco Molteni, Alessandra Pedrocchi

**Affiliations:** ^1^Nearlab@Lecco, Polo territoriale di Lecco, Politecnico di Milano, Lecco, Italy; ^2^Villa Beretta Rehabilitation Center, Valduce Hospital, Costa Masnaga, Italy; ^3^NearLab, Department of Electronics, Information and Bioengineering, Politecnico di Milano, Milan, Italy

**Keywords:** lower-limb exoskeleton, electromyography, automatic calibration, neurorehabilitation, therapy personalization

## Abstract

Stroke-related locomotor impairments are often associated with abnormal timing and intensity of recruitment of the affected and non-affected lower limb muscles. Restoring the proper lower limbs muscles activation is a key factor to facilitate recovery of gait capacity and performance, and to reduce maladaptive plasticity. Ekso is a wearable powered exoskeleton robot able to support over-ground gait training. The user controls the exoskeleton by triggering each single step during the gait cycle. The fine-tuning of the exoskeleton control system is crucial—it is set according to the residual functional abilities of the patient, and it needs to ensure lower limbs powered gait to be the most physiological as possible. This work focuses on the definition of an automatic calibration procedure able to detect the best Ekso setting for each patient. EMG activity has been recorded from Tibialis Anterior, Soleus, Rectus Femoris, and Semitendinosus muscles in a group of 7 healthy controls and 13 neurological patients. EMG signals have been processed so to obtain muscles activation patterns. The mean muscular activation pattern derived from the controls cohort has been set as reference. The developed automatic calibration procedure requires the patient to perform overground walking trials supported by the exoskeleton while changing parameters setting. The Gait Metric index is calculated for each trial, where the closer the performance is to the normative muscular activation pattern, in terms of both relative amplitude and timing, the higher the Gait Metric index is. The trial with the best Gait Metric index corresponds to the best parameters set. It has to be noted that the automatic computational calibration procedure is based on the same number of overground walking trials, and the same experimental set-up as in the current manual calibration procedure. The proposed approach allows supporting the rehabilitation team in the setting procedure. It has been demonstrated to be robust, and to be in agreement with the current gold standard (i.e., manual calibration performed by an expert engineer). The use of a graphical user interface is a promising tool for the effective use of an automatic procedure in a clinical context.

## Introduction

Stroke is the leading cause of long-term disability in adults despite the advances achieved in the management of its acute phase (Heiss and Kidwell, 2014; Tacchino et al., 2017). Independent walking in particular has been associated to an increase in patients' ability to perform daily life activities and self-esteem. Although more than half of patients achieve an independent walking, this achievement may not be functional to carry out activities of daily living. Locomotion is defined as a cyclical lower limbs activity that results from intricate dynamic interactions between a central program (at brain and spinal cord level) and feedback mechanisms from muscles, tendons, and skin afferences, as well as vision, audition, and vestibular senses (Rossignol et al., 2006). The lower limb neuromuscular pattern should compensate body weight support, provide forward and lateral stability, and forward progression to ensure intra and inter-limb multi-joints coordination (Perry and Burnfield, 2010). Common stroke-related locomotor impairments (e.g., imbalance, gait asymmetry, poor inter-limb coordination) are often associated with abnormal timing and intensity of recruitment of the affected and non-affected lower limb muscles.

Timing and intensity of muscles recruitment influence kinematic and kinetic pattern of lower limbs and intra and inter-limb coordination (Mulroy et al., 2003; Den Otter et al., 2007). Restoring the coordination in muscles activation of lower limbs is a key factor to facilitate recovery of gait capacity and performance, and to reduce maladaptive plasticity in stroke patients. Evidence within the last 20 years has shown that an injured central nervous system has the ability to reorganize after damage (Nudo, 2013; Gandolla et al., 2016). The reorganization is dependent on motor activity executed during rehabilitative training, and is followed by functional improvements (Edgerton et al., 2004; Maier and Schwab, 2006; Gandolla et al., 2014, 2016). In order to achieve better outcomes in stroke survivors, gait rehabilitation should target impairments in coordination and allow to augment the number of repetitions during walking practice (Eng and Tang, 2007).

Nowadays, wearable lower limbs powered exoskeletons may be a valuable adjunctive rehabilitation therapy aiming at augmenting training dose with repeatable, task-oriented, and controlled movements, as suggested by the principles of motor learning (Dietz and Harkema, 2004). In fact, as a common approach implemented in lower limbs exoskeleton commercial devices, the devices include actuators that support patient's legs through the gait cycle in the sagittal plane (e.g., Lokomat, Hocoma; ReWalk, ReWalk Robotics). The robotic device guides the legs through pre-programmed physiological gait patterns—this kind of therapeutic intervention is fairly new for stroke patients, however preliminary findings suggest that exoskeletal gait training is equivalent to traditional therapy for chronic stroke patients, while sub-acute patients may experience added benefit from exoskeletal gait training (Louie and Eng, 2016). Ekso is a wearable powered exoskeleton robot able to support stroke patients during over-ground gait training. The kinematic chain of the exoskeleton reproduces the human lower limbs walking pattern. In addition, Ekso actuators control patient's legs through the gait cycle in the sagittal plane. Ekso can be used as a therapeutic device in patients who must re-learn walking with a proper step pattern and functional weight shift by moving the patient's legs through a customizable predefined patient-tailored kinematic pattern. Ekso allows different setting for each patient in terms of swing velocity, step length, lateral shift. In this way, it is possible to control the walking pattern in terms of gait cycle timing (i.e., stance vs. swing phase duration), inter-limb and inter-joint coordination, lateral shift, trunk-lower limb angle, and timing to achieve appropriate limb loading.

The fine-tuning of the exoskeleton control system is crucial, and it is set according to the residual functional abilities of the patient. The interaction between exoskeleton and the patient can be seen under two different aspects: physical Human–Robot Interaction and cognitive Human–Robot Interaction (Pons, 2010; Lee et al., 2012). Physical Human–Robot Interaction includes the generation of supplementary forces to overcome human physical limits. In the case of the present study, the patient triggers each step, which however follows a predefined fully supported physiological trajectory. The interaction is therefore devoted to the generation of a proper gait cycle. Cognitive Human–Robot Interaction highlights the possibility to maintain the control of the robot from the human. In this study, the patient has the direct control on the trigger of each step though body lateral shift. Given the use of a commercial device, both aspects of Human-Robot Interaction depends on robotic device proper setting—the fine-tuning procedure is necessary to ensure the best power transfer between subject and robot. Surface ElectroMyoGraphy (sEMG) of the key muscles controlling multi-joints coordination of lower limbs is an effective way to non-invasively define motor control during spontaneous over-ground gait.

This work focuses on the definition of an automatic calibration procedure able to detect the best Ekso setting for each patient. Ekso setting has been defined using the neuromuscular pattern of the lower limbs collected with the superficial EMG in hemiparetic stroke patients. The proposed approach for an automatic calibration procedure is based on the hypothesis that the best Ekso setting yields to be best muscular activation as detected from superficial EMG electrodes, and that muscular activation is as better as closer to healthy controls muscular activation patter, particularly in terms of muscular activation timing.

## Materials and methods

### Experimental set-up

Patient's overground locomotion has been supported by Ekso (Ekso Bionics, Richmond, CA, USA). Ekso is a wearable bionic suit: it enables individuals with lower limb disabilities and minimal forearm strength to stand, sit and walk over a flat hard surface with a full weight-bearing reciprocal gait under the supervision of a physical therapist. Ekso is intended for non-ambulatory and ambulatory post-stroke patients, spinal cord complete, and incomplete injury patients with different etiology, and traumatic brain injury patients. It weighs 23 kg and can be used by individuals who weigh up to 100 kg and range in height from 160 to 190 cm. Patients must have a standing hip width at maximum of 43 cm. Ekso is equipped with four battery-powered motors at the hips and knees: these support or replace deficient neuromuscular function. There are four types of actuation for each patient step: (i) FirstStep, by which a physical therapist actuates steps with a button push; (ii) ActiveStep, by which the patient takes control of actuating steps via buttons on the crutches or walker; (iii) ProStep, by which the patient achieves the next step by moving body weight laterally and then forward; and (iv) ProStep Plus, by which steps are triggered by the user's lateral weight shift. The amount of power contribution to one or both legs during walking can be tuned with three types of assistance for each single step: (i) Bilateral Max Assist, in which Ekso provides full power to both legs and no strength is required from the patient; (ii) Adaptive Assist, in which patients with any amount of lower extremity strength contribute to their walking efforts and Ekso dynamically adjusts to produce a smooth, consistent gait; and (iii) Fixed Assist, where Ekso legs provide a fixed amount of pre-specified power to help patients to complete steps in a pre-defined amount of time. Within the present study, Ekso has been set with Prostep Plus, and Bilateral Max Assist. Ekso needs to be adjusted to fit patients' anthropometric data for a correct use of the device. In particular, it is necessary to collect hip width, length of right and left upper legs, and length of right and left lower legs.

The muscle activity has been recorded bilaterally with the FREEEMG wireless electromyograph (BTS Bioengineering, Garbagnate Milanese, Milano, Italy). Muscle groups considered for the analysis and placement of the electrodes has been selected accordingly to SENIAM guidelines (Hermens, 1999): tibialis anterior muscle (TA), soleus muscle (SOL), rectus femoris (RF), and semitendinosus muscle (SM). Lower limbs principal muscles have been selected for recording, and in particular, two couples of agonist/antagonist muscles in the proximal and distal compartment respectively, since they are more directly responsible for a correct walking-induced muscles activation profile, and EMG electrodes can be easily positioned without interfering with Ekso.

### Participants

Patients were recruited from the outpatient and inpatient services at the Villa Beretta Rehabilitation Centre (Costa Masnaga, LC, Italy). All patients had suffered from first-ever stroke, resulting in weakness of at least TA [to <4 on the Medical Research Council (MRC) scale Medical Research Council/Guarantors of Brain, 1986] and with a level of spasticity <2 as detected by Modified Ashworth Scale (Ansari et al., 2008) at hip, knee and ankle. Thirteen post-stroke patients were recruited [range: 29–74 years, mean (standard deviation): 52 (14)], comprising 10 male and 3 female subjects. Patient's characteristics along with the degree of functional recovery at the time of recruitment are listed in Table 1. The control group was composed of healthy volunteers with no neurological or orthopedic impairment. The healthy control group was aged between 21 and 49 years [mean (standard deviation): 36 (10) years], comprising four male and three female subjects. Experiments were conducted with approval from the Villa Beretta Rehabilitation Centre Ethics Committee and all subjects gave informed written consent in accordance with the Declaration of Helsinki.

**Table 1:** Patients characteristics.

**Patient ID**	**Age [years]**	**Sex [M/F]**	**Paretic side [R/L]**	**Stroke type [H/I]**	**FAC**	**Time from acute event [days]**
PT01	70	M	L	H	0	113
PT02	68	M	R	I	3	45
PT03	37	F	R	H	3	2,257
PT04	63	M	L	H	2	760
PT05	36	M	L	I	1	32
PT06	60	M	R	I	3	583
PT07	74	M	L	H	1	195
PT08	29	M	L	H	2	236
PT09	52	M	R	I	1	16
PT10	46	F	L	H	2	45
PT11	47	F	L	I	1	47
PT12	45	M	R	I	1	94
PT13	53	M	L	I	2	86

*M, male; F, female; L, left; R, right; H, hemorrhagic stroke; I, ischemic stroke; FAC, functional ambulatory category (Mehrholz et al., 2007)*.

### Current procedure for manual setting of Ekso

**Figure 1:**
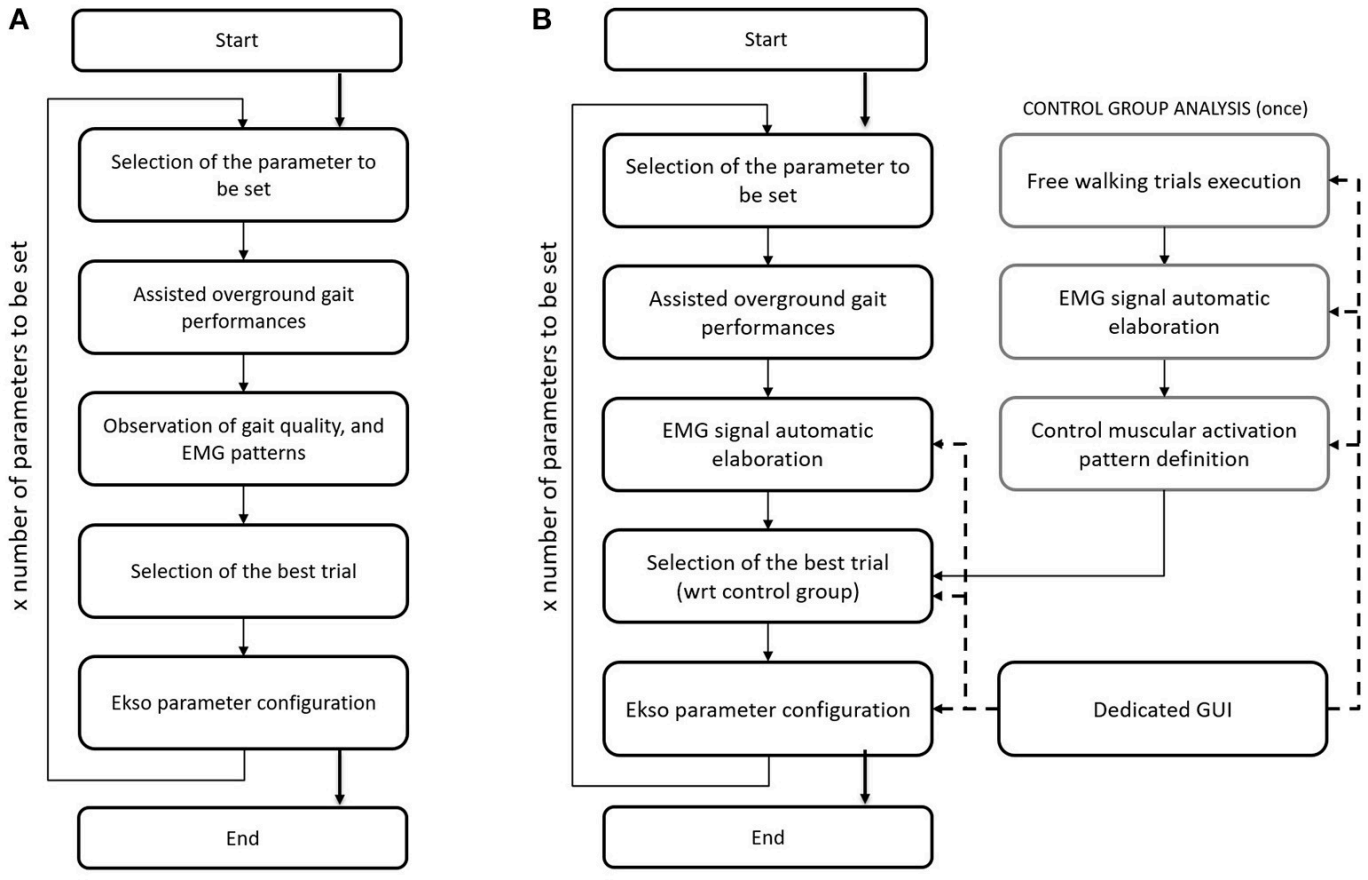
**(A)** Flow chart of the current Ekso manual calibration procedure. **(B)** Flow chart of the automatic Ekso calibration procedure.

Current gold standard for Ekso parameters setting in clinical environment (i.e., manual calibration) consists on the patient performing a series of overground walking trials with the values of tunable parameters changed by the rehabilitation team so to identify the best setting for the current patient and condition. These parameters are set on the basis of EMG signal derived from analyzed muscles, and by looking at patient gait. EMG signals are not processed in this case, and they are displayed on a laptop screen. The information drawn from raw EMG signals is muscles activation timing. The best activation timing for both healthy and paretic muscles is defined according to typical activity of major muscle groups during the gait cycle. In particular, the standard procedure includes the setting of the three main setting parameters, i.e., (i) lateral shift (displacement of body weight under the patient's foot); (ii) swing time; and (iii) step length. Manual calibration starts with the first parameter to be set (i.e., lateral shift). A series of overground gait trials are performed, while setting the parameter to different values. The gait trials are minimum three, where the default value, and higher and lower settings are tested. By means of observation of the gait quality, and EMG signals acquired during walking, the expert Ekso user along with the rehabilitation team selects the best parameter setting. The first parameter is then fixed, and the next parameters are considered in a recursive procedure until Ekso is properly set (Figure 1).

### Computational calibration procedure

The proposed approach for automatic computational calibration procedure is based on the same number of overground walking trials, and the same experimental set-up as in the current manual calibration procedure, where the observation of the gait quality, and EMG signals by the expert Ekso user is substituted by EMG signal computational analysis (Figure 1). EMG signal computational analysis is based on the hypothesis that muscular activation profile is as better as closer to healthy control population pattern. This is the reason why data from a representative group of control subjects were also collected. The computational calibration procedure is applied to healthy controls, and the non-paretic side of neurological patients. In fact, it is known that the more natural is the step of the unimpaired side, the more physiological is the gait, and the more it is possible to state that the global ambulation is close to normative.

#### Step identification procedure

**Figure 2:**
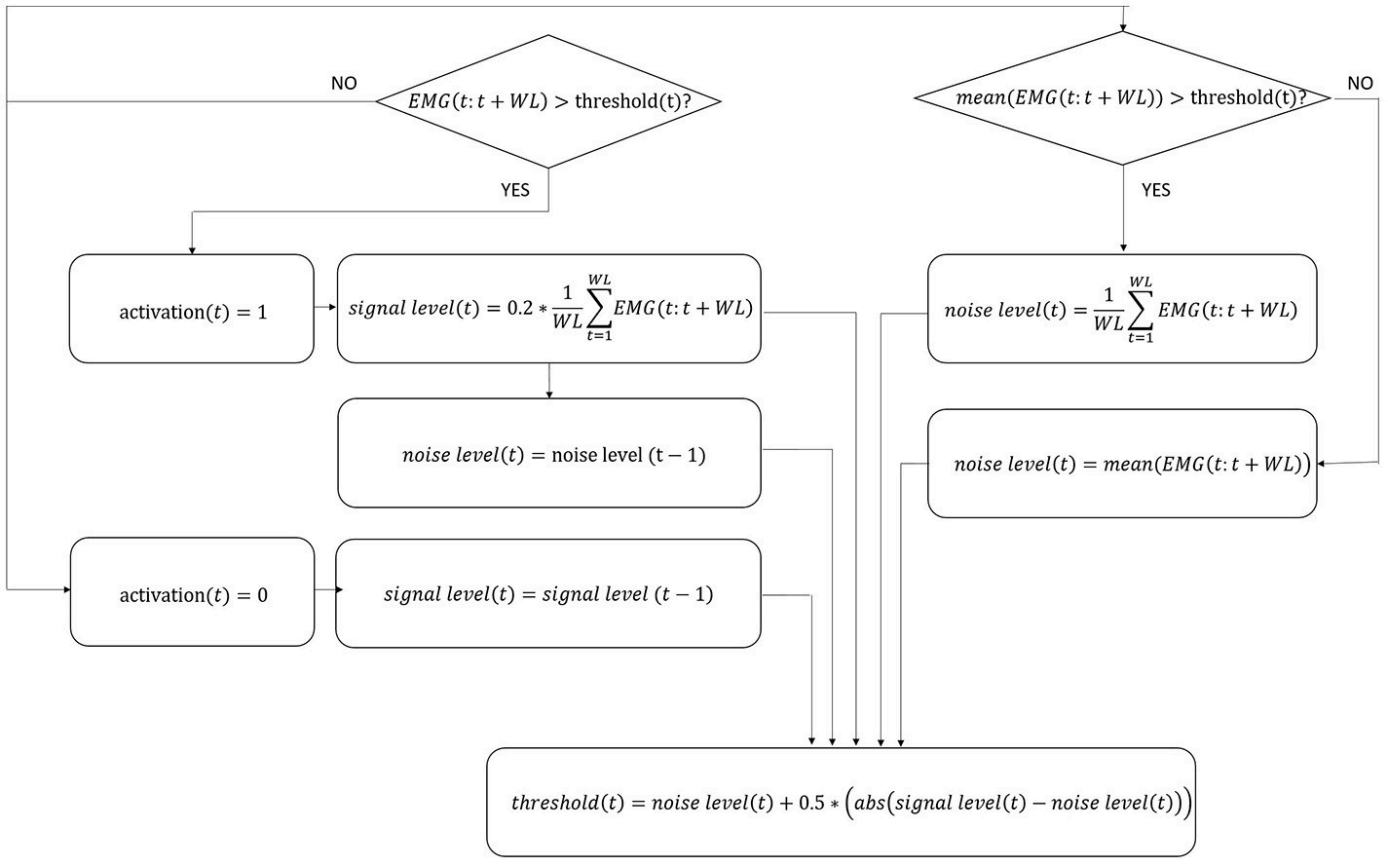
Flow chart of EMG signal activation/deactivation identification. The four variables in the algorithm, i.e., signal level, noise level, threshold, and activation, are updated following the equations indicated in figure for each sample t. WL, number of samples in the window; EMG, EMG signal; abs, absolute value.

Since it is currently impossible to autonomously extract data directly from Ekso sensors, it is not possible to synchronize Ekso with external systems (i.e., EMG). This limitation has been overcome by using a step identification procedure directly on the EMG signals. In particular, EMG signals coming from all muscular channels are pre-processed following a standard approach that includes high-pass filtering with a 6th order Butterworth filter at 20 Hz, rectification, and low-pass filtering with a 6th order Butterworth filter at 4 Hz (Solnik et al., 2008). Given that the computational calibration procedure should not include any additional workload to the rehabilitation team or to the patient, there are no footswitches or similar sensor available to give information about single steps. The proposed method is based on the hypothesis that the number of steps is proportional to the number of muscle activations. In order to satisfy this hypothesis, a mono-phasic muscle has been considered, so that only a single activation is expected throughout the step cycle. The Soleus muscle has been selected since is monophasic during the step (Pasinetti et al., 2013), i.e., it reaches only once the activation peak, characteristic which is preserved, as far as we observed, in our patients cohort. To this aim, Soleus EMG signal is further preprocessed to limit the bandwidth to frequencies where step cadence is located, i.e., 0–2 Hz (Pachi and Ji, 2005). Soleus de-activation is then identified through an algorithm based on a 20 samples sliding window and adaptive threshold derived from the integration of signal-to-noise ratio based adaptive threshold algorithm proposed by Sedghamiz, and Di Fabio and colleagues algorithm (Di Fabio, 1987; Sedghamiz, 2014). In particular, the algorithm is applied on the mean corrected EMG preprocessed signal, and the four variables—*signal level, noise level, threshold*, and *activation* (binary on/off variable used to describe muscle activation/deactivation)—are null at the beginning. Variables levels are dynamically calculated sample by sample as detailed in Figure 2, considering the EMG portion included within the 20 samples sliding window. The signal portion included between two Soleus muscle deactivation corresponds to a step cycle, starting from the end of the push-off phase. EMG signal of all considered muscles is segmented accordingly.

#### Muscular activation pattern definition

Muscular activation pattern for each muscle, both for control and for patients, is obtained by re-scaling each step to a 0–100% scale in terms of step duration, and afterwards by averaging all steps, and by normalizing the muscular step template in terms of amplitude with respect to the peak value for each trial. To obtain a healthy controls muscular pattern, all averaged muscular patterns resulted from five different trials performed per participant have been averaged. The accuracy of the signal segmentation technique has been evaluated through qualitative inspection of the morphology of the muscle activation profiles and through a quantitative analysis of the inter-step variability (i.e., coefficient of variation) to verify consistency with the muscular dynamics reported in literature (Winter and Yack, 1987). Finally, using the same onset/offset detection algorithm described to detect Soleus muscle deactivation (Figure 2), for each muscle an activation/deactivation profile is determined, where the information “the muscle is active or inactive” can be derived with respect to the percentage of the gait cycle (i.e., 0–100%).

#### Performance index extraction

So to define the best parameters setting, the Gait Metric index (GM) has been extracted from the healthy controls, and the non-paretic side of neurological patients. GM is an analytical combination of amplitude and activation timing (Ricamato and Hidler, 2005), and quantifies the deviation of the muscular activation pattern from normal ranges defined within the healthy control group.

In particular, GM is composed by the arithmetic mean between an amplitude, and a phase component determined through the comparison of each muscle activation pattern, and the correspondent healthy controls activation pattern. The amplitude component (AC) is obtained by summing the EMG values where both the patient and the healthy controls patterns are over or under threshold. In other words, for each given sample (i.e., 0–100% of the gait cycle), AC is increased if patient muscle is active when also healthy controls muscle is, or is inactive when also healthy controls muscle is inactive (Equation 1).

(1) $$\begin{array}{r} AC=\;\overset{100}{\underset{p=1}{\sum }}\left(HCAP\left(p\right)\right)\left(EMG\left(p\right)-threshold\right)\; \end{array}$$

Where *p* is the index representing gait cycle progression (i.e., 0–100%); HCAP is the Healthy Controls Activation Profile which is 1 for the healthy controls pattern active portions, and −1 for the inactive portions; *EMG(p)* is the patient EMG profile sample value; and *threshold* is the activation threshold defined as described in section Graphical User Interface (GUI) For Clinical Use.

AC is then normalized to obtain a value between 0 and 1 (AC_norm_) as follows (Equations 2–4).

(2) $$\begin{array}{lll} AC_{max} & = & \left(\left(1-threshold\right)*\;\#Active\right)\\ \;+\left(threshold\;*\;\#Inactive\right) \end{array}$$

(3) $$\begin{array}{rcl} AC_{min} & = & \;-1\;*\left(100-AC_{max}\right) \end{array}$$

(4) $$\begin{array}{rcl} AC_{norm} & = & \frac{AC-AC_{min}}{AC_{max}-AC_{\min}} \end{array}$$

Where #*Active* is the number of active samples in the healthy controls activation pattern; and #*Inactive* is the number of inactive samples in the healthy controls activation pattern.

The Phase Component (PC) is determined for each given sample (i.e., 0–100% of the gait cycle) by summing 1 if patient muscle is active when also healthy controls muscle is, or is inactive when also healthy controls muscle is, and 0 otherwise. PC is then normalized dividing the obtained value by 100.

Once the GM has been obtained for each considered muscle, a Weighted GM (WGM) is obtained by weighting each GM with the standard deviation of the correspondent muscle obtained in the healthy control group as follows (Equations 5, 6).

(5) $$\begin{array}{ll} Normalized\;St.\;Dev. & =\\ \left(\left[1\;1\;1\;1\right]-\frac{St.\;Dev.\;Healthy\;Sub.}{\sum_{i=1}^{n}{\left(St.Dev.\;Healthy\;Sub.\right)}_{i}}\right)*\frac{1}{n-1}. \end{array}$$

(6) $$\begin{array}{r} WGM=\overset{n}{\underset{i=i}{\sum }}GM_{i}^{*}{\left(Normalized\;St.\;Dev.\right)}_{i}. \end{array}$$

Where *n* represents the considered muscles, *St.Dev.HealthySub*. is the vector containing GM standard deviation obtained in the healthy controls group; *NormalizedSt.Dev*. is the normalized vector of standard deviations considered for GM weighting (i.e., sum equals 1).

The higher the WGM, the closer the performance is to the normative muscular activation pattern, in terms of both relative amplitude, and timing. The trial with the best WGM would correspond to the best parameter set.

### Computational calibration procedure validation

Repeatability of the automatic calibration procedure has been tested by running twice the algorithm for each participant (i.e., each neurological patient). The output parameters setting in the two runs have been compared through the Cohen's kappa for agreement between to evaluators (Cohen, 1960). The test of repeatability was important since the EMG signal portion selected to run the analysis is of free choice of the user, and therefore it cannot be taken for granted that the same steps are considered for the analysis. Indeed the computational procedure should be robust with respect to steps selection.

Manual calibration is performed by means of observation of the gait quality, and EMG signals acquired during walking. However, EMG signal analysis is only performed by sight on a non-processed signal, and it is therefore not reliable. To test this hypothesis, three different raters selected Ekso parameters setting only by inspecting non-processed EMG signal, without seeing the patients. The agreement between the different raters has been evaluated through Fleiss' Kappa (Landis and Koch, 1977).

In addition, the agreement between computational calibration procedure parameters setting, and the gold standard procedure (i.e., parameters set by the expert clinical engineer and rehabilitation team during the effective calibration session) has been evaluated by Cohen's kappa (Cohen, 1960; Gandolla et al., 2015).

### Graphical user interface (GUI) for clinical use

**Figure 3:**
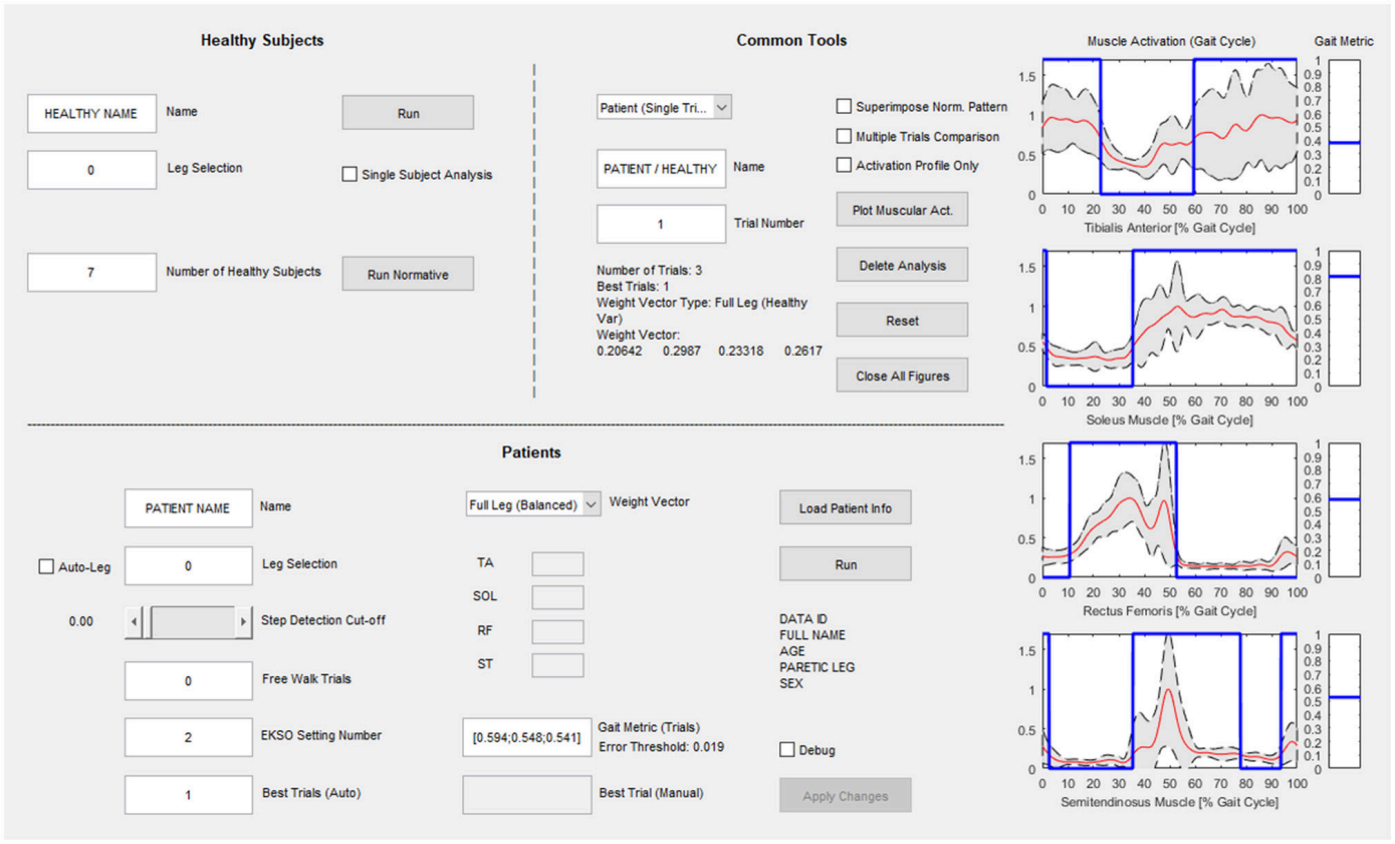
Graphical User Interface for clinical use. The “Healthy Subjects” section is dedicated to the analysis of healthy subjects and the calculation of the normative muscular activation pattern; the “Patients” section is dedicated to patient data analysis and searching for the best Ekso configuration; in the “Common Tools” section the user can perform an additional analysis of patients or healthy subject's data.

The computational calibration procedure has been implemented in a custom-made and guided software developed in MATLAB environment (Figure 3) to support the use of the proposed approach in clinical practice. The interface has three sections: the “Healthy Subjects” section dedicated to the analysis of healthy controls and the calculation of the normative muscular activation pattern; the “Patients” section for patient data analysis and searching for the best Ekso GT configuration; the “Common Tools” section where the user can perform an additional analysis of patients or healthy controls data.

## Results

### Muscular activation pattern

**Figure 4:**
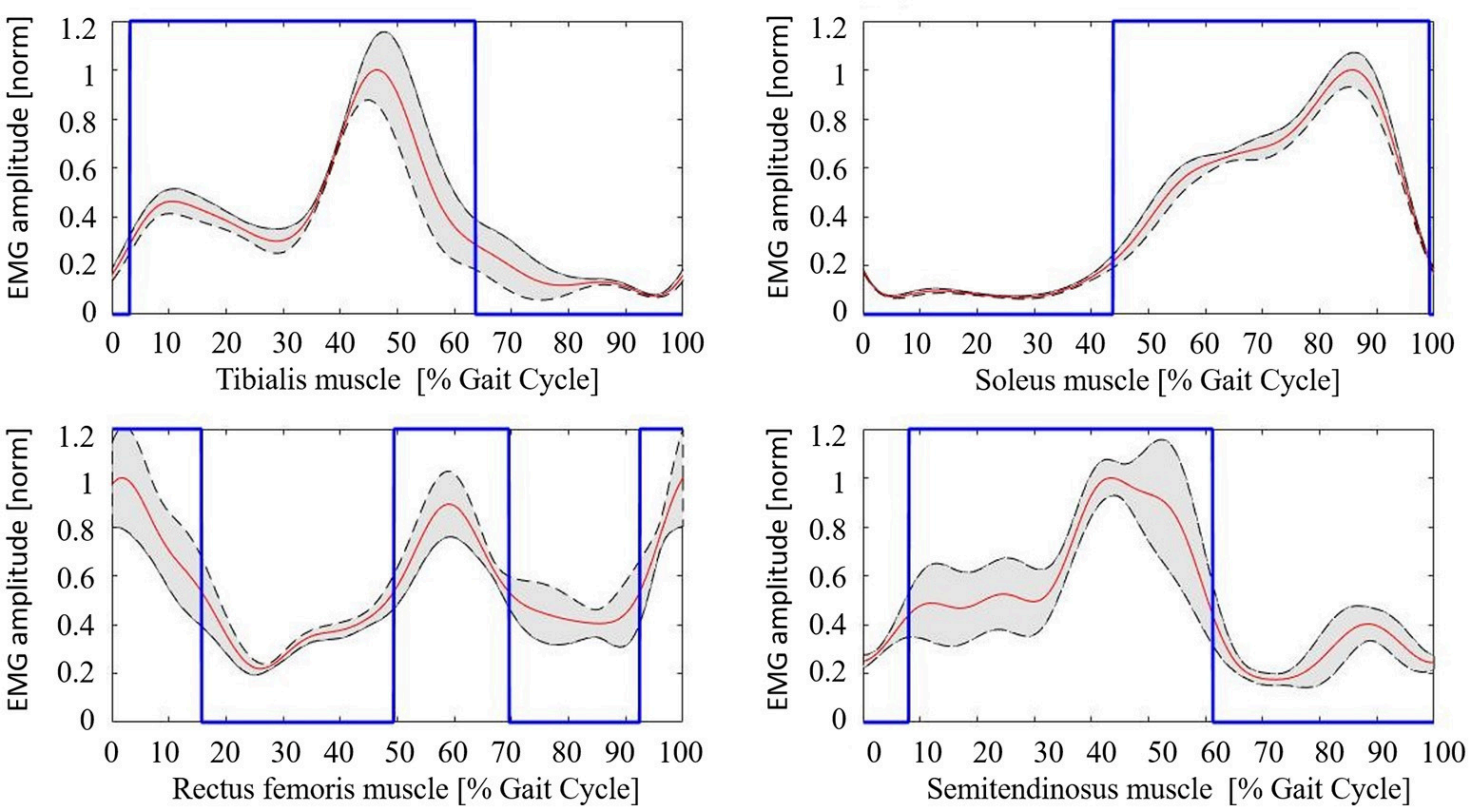
Healthy controls muscular pattern. Red line, mean; gray shaded area, standard deviation; blue line, activation profile (active/non-active muscle window).

The healthy controls muscular pattern is shown in Figure 4. The qualitative inspection of the morphology of the muscle activation profiles reflects what has been found in literature evidences (Winter and Yack, 1987; Tao et al., 2012), and in particular:

- Tibialis anterior (TA) muscle is active to prevent contact of the toes with the ground during the initial and intermediate swing phase (0–30%); an activation peak happens during the terminal and load acceptance phases (30–45% GC). TA activity is reduced during the stance phase.
- Soleus muscle (SOL) activity starts in the load acceptance phase (35–45% GC), increases in intermediate support phase (45–65% GC), and then reaches its peak during pre-oscillation phase (75–85% GC). When the push-off phase is complete, the soleus muscle remains inactive throughout the swing phase.
- Rectus femoris (RF) muscle has moderate activity in the early oscillation phases (0–10%), so it reaches an activation peak in the acceptance phase and intermediate support phase acting as a stabilizer (30–65% GC). There is a final activation in the propulsion and lifting phase of the limb (75–100% GC).
- Semitendinosus muscle (ST) has moderate activity in the early swing phase (0–10% GC), then achieves a peak in the terminal oscillation and acceptance phases aiming at stopping the movement of the limb (30–45%). Its activity is slowly reduced during the intermediate support phase.

The mean value of the coefficient of variation across all subjects for the considered muscles are 0.230 (TA), 0.167 (SOL), 0.369 (RF), and 0.365 (ST), respectively.

**Figure 5:**
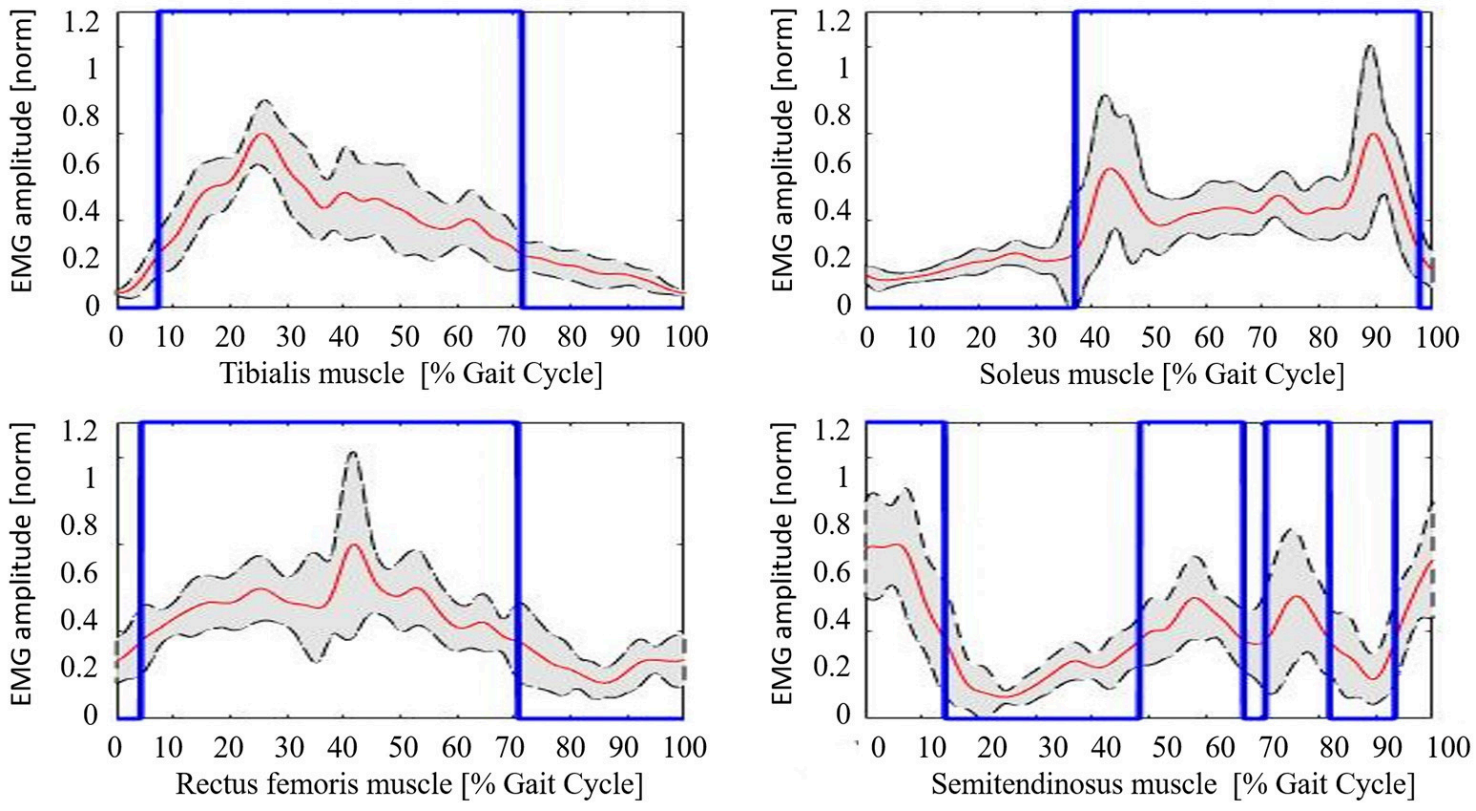
Example of neurological patient muscular pattern (PT11). Red line, mean; gray shaded area, standard deviation; blue line, activation profile (active/non-active muscle window).

Neurological patients muscular activation pattern is quite different among subjects, as expected (an example is shown in Figure 5). For all patients, steps segmentation has been successfully performed on the non-paretic EMG signal during robotic-assisted gait trials, and the gait metric index has been calculated for all trials, and all patients.

### Gait metric behavior

**Figure 6:**
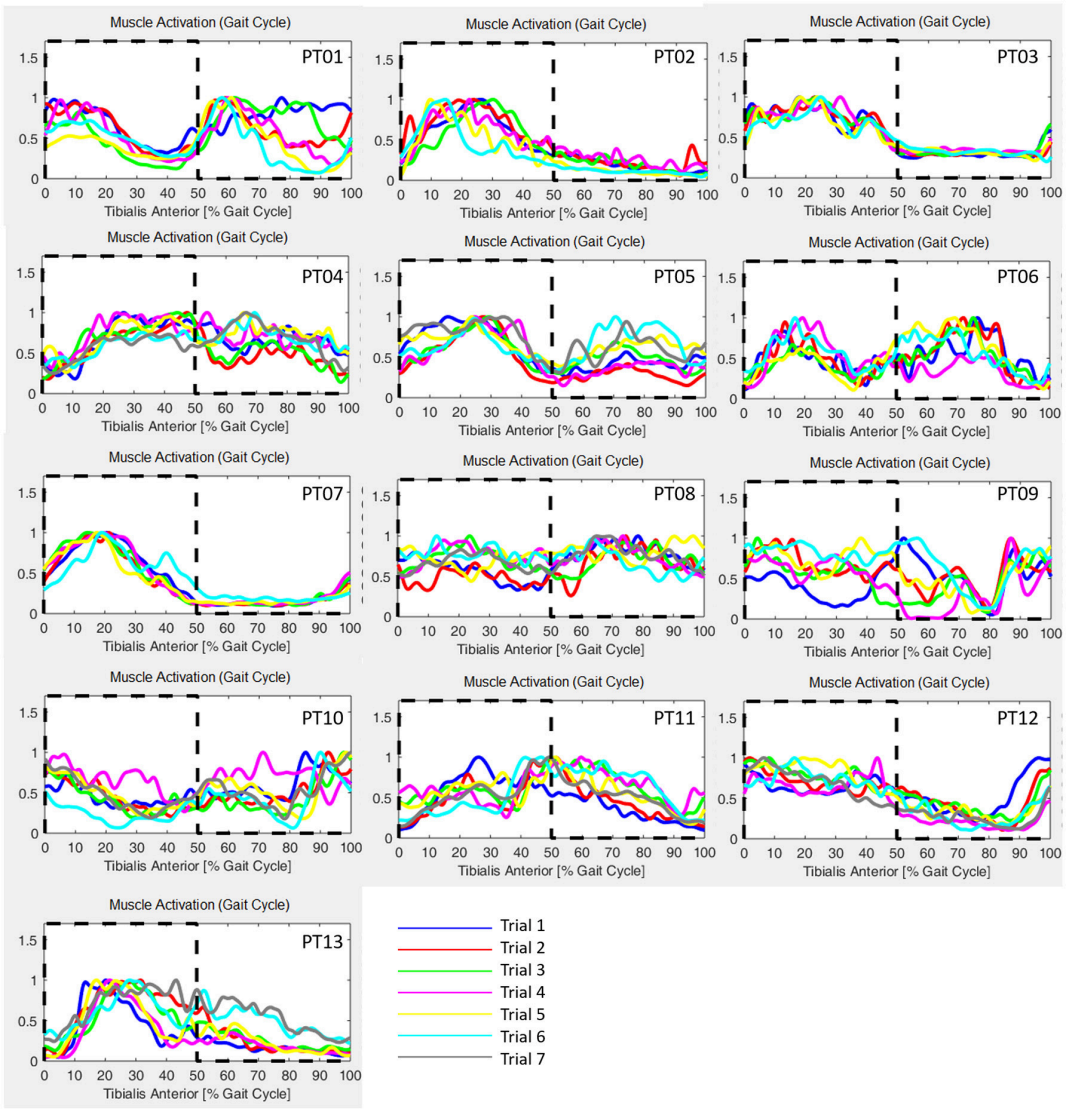
Tibialis Anterior muscle EMG profiles for all patients and all performed trials. Dashed line: healthy controls activation window.

GM has been calculated for each trial of the healthy controls cohort, and results are reported in Table 2, and are in accord with values reported in literature for a walking speed obtained during spontaneous walking, i.e., 1.3–1.6 m/s (Ricamato and Hidler, 2005). To check for WGM variability, WGM has been calculated for each control participant for the five walking trials obtaining standard deviations equal to 0.007, 0.014, 0.023, 0.031, 0.027, 0.005, and 0.026 respectively. Table 3 shows the detailed WGM scores obtained by patients in the seven walking trials with different Ekso parameters settings. As it can be observed, in some cases, WGM differences between alternative parameters settings are not crucial (i.e., difference lower that healthy controls cohort mean WGM standard deviation−0.019). In these cases, Ekso parameters setting is therefore not crucial in terms of EMG activations obtained. WGM variability is higher within the first three trials, correspondent to the selection of first parameter, i.e., lateral shift, while decreases in the other trials. As a representative muscle, Tibialis Anterior EMG mean profile for each trial and each patient is represented in Figure 6. As it can be observed, some of the patients present quite substantial differences in terms of muscles activity, which can be easily detected by sight (e.g., PT01 or PT13). Other patients (e.g., PT07) shows muscles activity profiles almost superimposable among trials. Again, Ekso parameters setting is particularly crucial for patients who present substantially different muscles activation profiles.

**Table 2:** Gait Metric index values obtained by the healthy subjects cohort.

**Subject**	**TA**	**SOL**	**RF**	**ST**
S01	0.591	0.799	0.634	0.704
S02	0.772	0.819	0.709	0.717
S03	0.797	0.847	0.738	0.786
S04	0.612	0.784	0.641	0.744
S05	0.747	0.847	0.624	0.763
S06	0.848	0.843	0.741	0.812
S07	0.716	0.843	0.533	0.654
Mean	0.726	0.826	0.660	0.740
Std dev	0.095	0.026	0.075	0.054

*TA, tibialis anterior muscle; SOL, soleus muscle; RF, rectus femoris; SM, semitendinosus muscle; Std dev, standard deviation*.

**Table 3:** Weighted Gait Metric index values obtained by the patients cohort.

	**Trial 1**	**Trial 2**	**Trial 3**	**Trial 4**	**Trial 5**	**Trial 6**	**Trial 7**
PT01	0.5353	0.5435	0.5184	0.5689	0.5807	0.5989	–
PT02	0.6402	0.6263	0.6144	0.6305	0.6774	0.6744	–
PT03	0.5738	0.5796	0.5848	0.5772	0.5868	0.5890	–
PT04	0.5410	0.5565	0.5804	0.5229	0.5275	0.5443	0.5319
PT05	0.5842	0.5854	0.5379	0.5972	0.5372	0.5359	0.5815
PT06	0.5241	0.4980	0.5007	0.5555	0.5044	0.5485	–
PT07	0.5862	0.5927	0.6119	0.6097	0.5658	0.5960	–
PT08	0.4960	0.4983	0.5160	0.5555	0.5013	0.5452	0.5099
PT09	0.4784	0.5048	0.5100	0.5694	0.5241	0.5278	–
PT10	0.5235	0.5187	0.5079	0.4960	0.5007	0.4808	0.5471
PT11	0.5860	0.5538	0.5857	0.5626	0.6214	0.5978	0.6414
PT12	0.5915	0.5984	0.6017	0.6271	0.6268	0.6257	0.6175
PT13	0.6347	0.7117	0.6926	0.6268	0.6523	0.6598	0.6930

### Reproducibility and validity of computational calibration

The computational calibration procedure is robust with respect to steps selection, as shown by Cohen's kappa equals to 0.883, i.e., strong agreement (Sim and Wright, 2005). On the other side, the agreement between the three different raters who selected Ekso parameters setting only by inspecting non-processed EMG signal is equal to 0.296, i.e., mediocre agreement. Agreement between computational calibration procedure, and gold standard (i.e., expert engineer setting) shows substantial agreement, with Cohen's kappa equals to 0.648, while agreement between the three different raters who selected Ekso parameters setting only by inspecting non-processed EMG signal and gold standard is very weak with Cohen's kappa equal, respectively to 0.095, 0.058, and 0.045 (Sim and Wright, 2005). In particular, for the three Ekso parameters Cohen's kappa for the agreement between automatic procedure and gold standard selection are 0.614, 0.591, and 0.780, respectively.

## Discussion

Gait recovery in post-stroke patients is one of the main goals of post-stroke rehabilitation (Molteni et al., 2017). Literature evidences demonstrated that central nervous system can reorganize after injury and that reorganization depends on motor activity performed during rehabilitative training (Edgerton et al., 2004; Maier and Schwab, 2006). Wearable robotic exoskeleton may be intended like an external environment acting with the patient—an extension of the body of the patient. Robotic devices induce patients' lower limbs to complete a pre-defined motor pattern according to a pre-programmed kinematic profile allowing subjects with gait dysfunctions to perform an over-ground gait training based on the principle of motor relearning.

There is a paucity of published data on powered robotic exoskeletons for gait rehabilitation in post-stroke patients. In a recent review (Louie and Eng, 2016) on the use of wearable powered exoskeletons in stroke patients, authors describe studies in which different robotic devices were used on a small number of stroke patients without general consensus on the results. Molteni et al. (2017) performed a pre-post study to analyse the effects of a wearable powered exoskeleton on 23 sub-acute and chronic stroke patients. Authors claimed that it is possible to modify clinical outcome measures in sub-acute and chronic post-stroke patients after 12 sessions of gait training with a powered wearable robotic exoskeleton after fine-tuning of the kinematic gait cycle parameters. The fine-tuning of wearable robotic device parameters is therefore essential to produce the best neuromuscular pattern of the lower limbs enhancing short-term neuromodulation. This may be a way to induce long-term potentiation of the mechanism controlling the gait pattern of non-affected and affected side (Kwakkel et al., 1999).

For this reason, the use of an automatic calibration procedure to identify the best settings for each patient is very important. This approach, which is repeatable, robust, and based on quantitative measures, may underline aspects hardly detectable only through direct observation, and may provide a valuable support to the rehabilitation team.

In this work, an automatic calibration procedure has been proposed for Ekso. The proposed approach is based on the hypothesis that the best Ekso setting yields to a muscular activation as close as possible to healthy controls muscular activation pattern, given that restoring a correct activation pattern is a key aspect of the rehabilitation program of neurological patients (Zhang et al., 2017). Although the sample size of healthy subjects is limited, the derived muscular activation patterns for all muscles agree with those reported in literature (Ricamato and Hidler, 2005). Coefficients of variations show the same relationship among muscles as described in literature (Winter and Yack, 1987). Distal muscles (i.e., TA and SOL muscles) present lower coefficients of variations with respect to proximal lower limbs muscles (i.e., RF and SM muscles). This is in line with the role of proximal muscles during gait, which is of support and equilibrium control. Given in fact the complexity of their functions, proximal muscles activation profile results to be more variable among successive steps (Winter and Yack, 1987). As a clinical recommendation for the computational calibration procedure everyday use, the authors suggest if possible to acquire the EMG signal from all four principal leg muscles or, as a possible alternative, to register distal muscles activity. In fact, as previously introduced, the function of proximal muscles during gait might be identified in the maintenance of balance, which is a complex motor task in post-stroke patient and the disability due to the paretic limb introduces compensatory mechanisms that affect its performance. Typically, if excessive co-contraction of distal muscles occurs, compensation is performed at the proximal level (Higginson et al., 2006). An analysis only based on the activity of the semitendinosus and rectus femoris muscles cannot guarantee the optimum performance of the motor task because a physiological activation of the proximal muscles may correspond to an abnormal activation of the distal muscles. Conversely, an analysis of both the soleus and Tibialis Anterior muscle seems to be more effective in defining the Ekso settings because a proper distal activation pattern more likely corresponds to a non-compensatory activation of proximal muscles.

The goal of the proposed approach is to equip the clinician with an instrument that could help clinician to identify the best Ekso setting, singularly for each patient. As far as we know, there are any quantitative data published in literature or indications provided by the fabricant so to evaluate the correctness of the setting. The gold standard procedure is manual regulation by expert operators, and, as it can be observed by the poor agreement revealed by Cohen's kappa between different operators in selecting best Ekso parameters, it lays on subjective evaluation, and it is not repeatable. The automatic procedure selection has been compared to the setting selection of an expert operator in the Villa Beretta Rehabilitation Center, as suggested by Ekso Company itself, and obtained substantial agreement, being at the same time robust for different steps selections. In this case, we are not claiming we are obtaining better results with respect to manual calibration in terms of best setting, but we are claiming that the automatic procedure is robust and repeatable with respect to a gold standard, and can be used by any operator.

The proposed approach allows supporting the rehabilitation team in the setting procedure, and it has been demonstrated to be robust, and to be in agreement with the current gold standard. The use of a graphical user interface is a promising tool for the effective use of an automatic procedure in a clinical context. Indeed, the automatic calibration procedure does not imply any additional workload for the patient or the therapist with respect to the manual calibration procedure. The automatic calibration procedure has been validated with respect to the current gold standard, which is the selection of the expert Ekso user. However, the use of the automatic calibration procedure may allow a correct parameters setting from the very beginning of Ekso use, also when the rehabilitation team is still not well trained. The identification of an automatic procedure able to detect in an objective way the best devices setting, allows to plan a completely new individual tailored rehabilitation strategy.

## Author contributions

MG, EG, FM, and AP: Conceived the design of the work; EG and GC: Acquired the data; MG, EG, AD, and AP: Conceived the algorithm and analyzed the data; All authors contributed in interpreting data for the work. MG, EG, and AD: Drafted the manuscript; AP, GC, and FM: Revised it critically for important intellectual content. All authors read and approved the final version of the manuscript.

### Conflict of interest statement

The authors declare that the research was conducted in the absence of any commercial or financial relationships that could be construed as a potential conflict of interest.
